# Impact of Eating Speed on Muscle Mass in Older Patients With Type 2 Diabetes: A Prospective Study of KAMOGAWA–DM Cohort

**DOI:** 10.3389/fnut.2022.919124

**Published:** 2022-06-23

**Authors:** Genki Kobayashi, Yoshitaka Hashimoto, Fuyuko Takahashi, Ayumi Kaji, Ryosuke Sakai, Takuro Okamura, Hiroshi Okada, Noriyuki Kitagawa, Naoko Nakanishi, Saori Majima, Takafumi Osaka, Takafumi Senmaru, Emi Ushigome, Mai Asano, Masahide Hamaguchi, Masahiro Yamazaki, Michiaki Fukui

**Affiliations:** ^1^Department of Endocrinology and Metabolism, Graduate School of Medical Science, Kyoto Prefectural University of Medicine, Kyoto, Japan; ^2^Department of Diabetes and Endocrinology, Matsushita Memorial Hospital, Moriguchi, Japan; ^3^Department of Diabetology, Kameoka Municipal Hospital, Kyoto, Japan; ^4^Department of Endocrinology and Diabetes, Ayabe City Hospital, Ayabe, Japan

**Keywords:** eating speed, diet, muscle mass, diabetes, sarcopenia, older patients

## Abstract

**Background and Aims:**

Maintenance of muscle mass is important for sarcopenia prevention. However, the effect of eating speed, especially fast, normal, or slow speed, on muscle mass changes remains unclear. Therefore, the purpose of this prospective study was to investigate the effect of eating speed on muscle mass changes in patients with type 2 diabetes (T2DM).

**Methods:**

This study included 284 patients with T2DM. Based on a self–reported questionnaire, participants were classified into three groups: fast–, normal–, and slow–speed eating. Muscle mass was assessed using a multifrequency impedance body composition analyzer, and skeletal muscle mass (SMI) decrease (kg/m^2^/year) was defined as [baseline SMI (kg/m^2^)–follow–up SMI (kg/m^2^)] ÷ follow–up duration (year). The rate of SMI decrease (%) was defined as [SMI decrease (kg/m^2^/year) ÷ baseline SMI (kg/m^2^)] × 100.

**Results:**

The proportions of patients with fast–, normal–, and slow–speed eating were, respectively, 50.5%, 42.9%, and 6.6% among those aged <65 years and 40.4%, 38.3%, and 21.3% among those aged ≥65 years. In patients aged ≥65 years, the rate of SMI decrease in the normal (0.85 [95% confidence interval, CI: −0.66 to 2.35]) and slow (0.93 [95% CI −0.61 to 2.46]) speed eating groups was higher than that in the fast speed eating group (−1.08 [95% CI −2.52 to 0.36]). On the contrary, there was no difference in the rate of SMI decrease among the groups in patients aged <65 years. Compared with slow speed eating, the adjusted odds ratios of incident muscle loss [defined as rate of SMI decrease (%) ≥0.5%] due to fast– and normal–speed eating were 0.42 (95% CI 0.18 to 0.98) and 0.82 (95% CI 0.36 to 2.03), respectively.

**Conclusion:**

Slow–speed eating is associated with a higher risk of muscle mass loss in older patients with T2DM.

## Introduction

With increase in the number of patients with type 2 diabetes mellitus (T2DM), the number of elderly patients with T2DM has also increased worldwide (1). In elderly patients with DM, insulin signals are reduced because of insulin resistance and reduced insulin secretion, leading to increased protein degradation and decreased protein synthesis, ultimately reducing muscle mass (2). Because muscle is a major organ that accounts for 20% of the body's total glucose metabolism (3); muscle mass is very important for patients with T2DM. Loss of muscle mass leads to sarcopenia, which is known to increase the risk of not only cardiovascular disease (4, 5) but also mortality (6–8). Moreover, the prevalence of sarcopenia is high in patients with T2DM (9, 10).

Sarcopenia can be prevented through modifying eating habits. Previous studies have shown that adequate macro– and micronutrients are important for maintaining muscle mass in patients with T2DM (11–13). Eating speed is also important because fast eating is associated with obesity (14), non–alcoholic fatty liver disease (NAFLD) (15), and T2DM (16); therefore, slow eating speed may be effective for lowering obesity risk. On the contrary, slow eating increases the risk of undernutrition (17). It is because slow speed eating increases anorexigenic gut hormones such as peptide YY and glucagon–like peptide−1 (GLP−1) (18, 19) and diet–induced thermogenesis (20, 21). Additionally, we recently conducted a cross–sectional study and reported that there is an association between slow eating speed and a risk of sarcopenia in elderly patients with T2DM (22). However, it is not clear which eating speed (fast, normal, or slow) is effective for maintaining muscle mass in patients with T2DM. Therefore, we conducted a prospective cohort study with an aim to clarify the effect of various eating speed and changes in muscle mass in older patients with T2DM.

## Materials and Methods

### Study Participants

The KAMOGAWA–DM cohort study is an ongoing prospective cohort study that began in 2014 (23). This study aimed to clarify the natural clinical history of patients with T2DM. Patients also have chronic diseases other than diabetes such as hypertension and dyslipidemia. Hypertension was defined as systolic blood pressure ≥140 mmHg, diastolic blood pressure ≥90 mmHg and/or taking anti–hypertensive drugs, and dyslipidemia was defined as low–density lipoprotein cholesterol ≥140 mg/dL, triglycerides ≥150 mg/dL and/or taking anti–dyslipidemia drug. This study enrolled diabetes specialty outpatients of university hospital (Kyoto Prefectural University of Medicine (KPUM) Hospital, Kyoto, Japan) and city hospital (Kameoka Municipal Hospital, Kameoka, Japan) with written informed consent. This study was performed in accordance with the Declaration of Helsinki and was approved by the ethics committee of KPUM (No. RBMR–E−466–5). Patients who correctly answered the Brief–Type Self Diet History Questionnaire (BDHQ) between January 2016 and April 2018 were extracted to this study. Patients who had the following criteria were excluded: no T2DM, missing data of eating speed or body impedance analyzer (BIA) parameters, extremely high or low energy intake (>4000 or <600 kcal/day) because of unreliability (24), follow–up duration of <6 months, steroid usage, and no follow–up.

### Data Collection

We used a standardized questionnaire to collect data on the duration of T2DM, exercise, and smoking. We defined exercisers as those who performed physical activities at least once a week regularly, and we divided the participants into exercisers and non–exercisers. The questionaries about smoking consisted of a question “ do you smoke?” and the 2 choices, “yes” or “no”. Then, we classified “yes” and ”no” as “smoker”, and “non–smoker”. Medication data, including information on steroids, insulin and sodium glucose cotransporter−2 (SGLT2) inhibitors, were collected from the medical records.

Samples of venous blood were collected from participants after overnight fasting, and levels of fasting plasma glucose (FPG) and hemoglobin A1c (HbA1c) were measured from these blood samples.

### Body Composition Measurement

Evaluation of the body composition of patients was performed by a multifrequency BIA (InBody 720; InBody Japan, Tokyo, Japan), which was reported to correlate well with the results of dual–energy X–ray absorptiometry (DEXA) (25). Body weight (kg) and appendicular muscle mass (kg) were measured, and the body mass index (BMI, kg/m^2^) and skeletal muscle mass index (SMI, kg/m^2^) were calculated, which were defined, respectively, as body weight (kg) ÷ height–squared (m^2^) and appendicular muscle mass (kg) ÷ height–squared (m^2^). Next, ideal body weight (IBW) was estimated using the following formula: 22 × height–squared (m^2^) (26). Furthermore, definition of SMI decrease (kg/m^2^/year), the rate of SMI decrease (%) were follows; [baseline SMI (kg/m^2^)–follow–up SMI (kg/m^2^)] ÷ follow–up duration (year) and [SMI decrease (kg/m^2^/year) ÷ baseline SMI (kg/m^2^)] × 100. Loss of muscle mass was defined as the rate of SMI decrease (%) ≥0.5%, because it was reported that muscle mass decreases with aging; in particular, the average rate of loss in aged people was 0.5%−1% per year (27).

### Assessment of Habitual Food, Nutrient Intake, and Eating Speed

Habitual food intake, nutrient intake, and eating speed were assessed using the BDHQ, which estimated the dietary intake of 58 food and beverage items during the past month. BDHQ is a dietary intake questionnaire for Japanese and has been validated. Previous studies showed that the correlation between BDHQ and semi–weighed dietary records was *r* = 0.44 to 0.56 (28–31). From the BDHQ, we gathered data on total energy intake (kcal/day), protein intake (g/day), fat intake (g/day), and carbohydrate intake (g/day). Total energy intake (kcal/IBW/day) was calculated as total energy intake (kcal/day) ÷ IBW. Protein intake (% energy), fat intake (% energy), and carbohydrate intake (% energy) were calculated as [protein intake (g/day) × 4 (kcal/g) ÷ total energy intake (kcal/day)] × 100, [fat intake (g/day) × 9 (kcal/g) ÷ total energy intake (kcal/day)] × 100, and [carbohydrate intake (g/day) × 4 (kcal/g) ÷ total energy intake (kcal/day)] × 100, respectively. Alcohol intake was also estimated, and habitual alcohol intake was defined as alcohol intake of >30 g/day for men and >20 g/day for women (32).

The questionaries about eating speed consisted of a question “how fast do you eat?” and the 5 choices, “very fast” “a little fast” “normal” “a little slow” or “very slow”. Then, “very fast”, or “a little fast”, “normal”, and “a little slow” or “very slow” were classified as “fast speed eating”, “normal speed eating”, and “slow speed eating”(15).

### Statistical Analysis

Continuous variables are presented as mean (standard deviation [SD]), and categorical variables are presented as number (%). *P*–value of <0.05 was considered to indicate statistical significance. The between–group differences were evaluated using one–way analysis of variance and the Tukey–Kramer test or chi–square test. We analyzed patients aged <65 years and patients aged ≥65 years separately because the recommended dietary energy intake varied by age (33).

Multiple regression analyses were performed to calculate the odds ratio (OR) and 95% confidence interval (CI) and assess the effects of eating speed on SMI decrease (kg/m^2^/year), rate of SMI decrease (%), and logistic regression analyses was also carried out to calculate the OR and 95% CI for eating speed on incident muscle mass loss. Factors of age, sex, HbA1c (%), insulin usage, SGLT2 inhibitor use, smoking, exercise, alcohol consumption, total energy intake (kcal/IBW/day), protein intake (% energy), and BMI, were considered to be independent variables.

In addition, we used the other cutoff levels for loss of muscle mass: SMI decrease of ≥1.2% (34) and 2.0% (35).

All statistical analyses were performed using JMP ver13.2 software (SAS Institute Inc., Cary, NC, USA).

## Results

**Figure 1:**
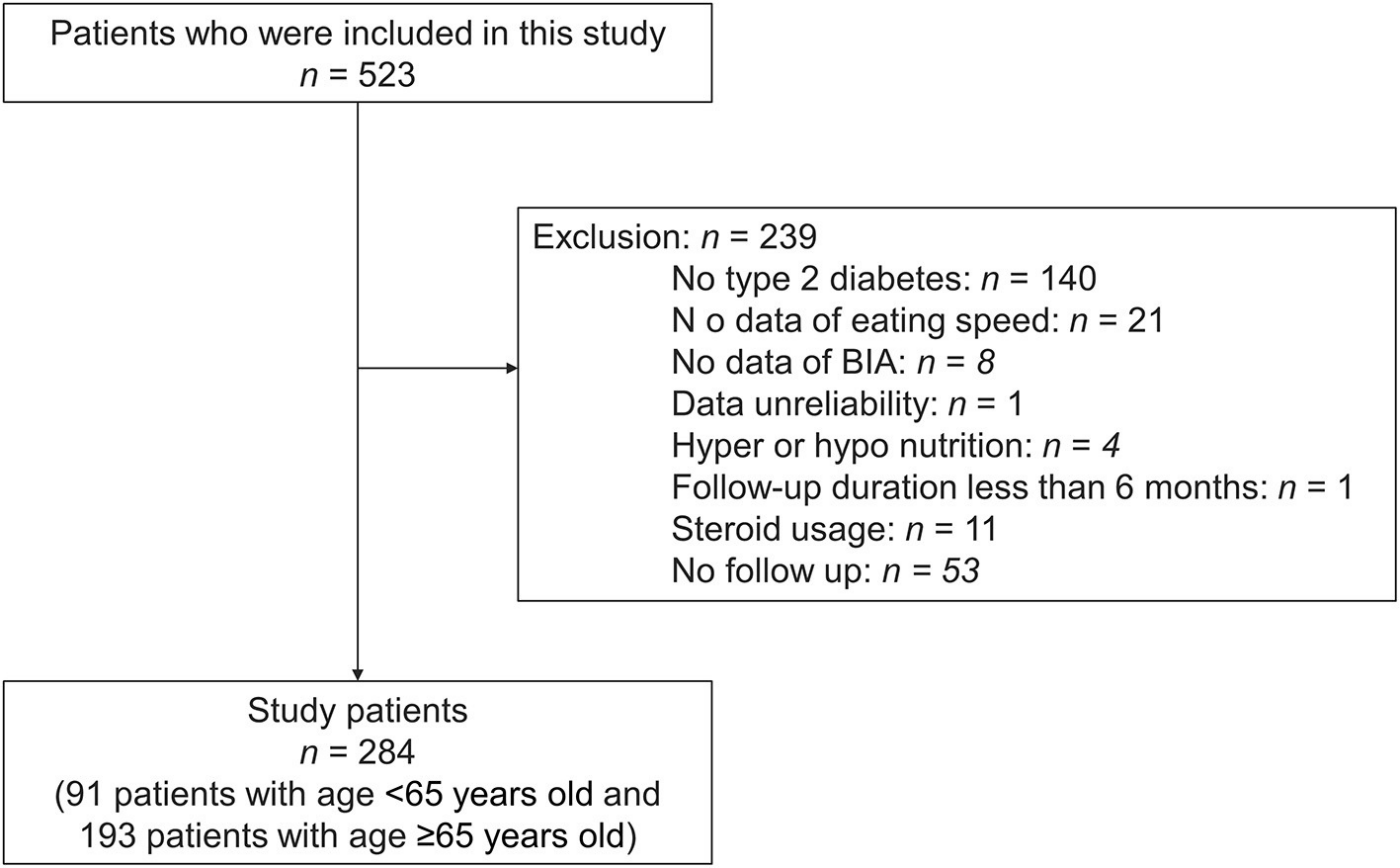
Inclusion and exclusion flow. BIA, body impedance analyzer.

A total of 523 patients participated in the study; however, 239 participants were excluded for various reasons and 284 participants were finally included in the study (Figure 1).

The participants' characteristics are shown in Table 1. In patients aged <65 years, mean BMI and SMI were 26.6 kg/m^2^ and 7.3 kg/m^2^, respectively. Furthermore, the percentages of patients with fast, normal, and slow eating were 50.5%, 42.9%, and 6.6%, respectively. In patients aged ≥65 years, mean BMI and SMI were 23.8 kg/m^2^ and 6.9 kg/m^2^, respectively, and the proportions of patients with fast, normal, and slow speed eating were 40.4%, 38.3%, and 21.3%.

**Table 1:** Clinical characteristics of study participants.

**Age <65 years old**	**All** ***n*** **= 91**	**Fast,** ***n*** **= 46**	**Normal, *n* = 39**	**Slow,** ***n*** **= 6**	***p***
Age, years	54.0 (8.7)	53.4 (10.0)	54.4 (7.4)	55.3 (6.3)	0.806
Men, *n* (%)	44 (48.4)	23 (50.0)	19 (48.7)	2 (33.3)	0.743
Duration of diabetes, years	9.2 (6.8)	9.8 (7.0)	8.7 (6.7)	7.3 (6.6)	0.615
Family history of diabetes, *n* (%)	52 (57.1)	25 (54.4)	22 (56.4)	5 (83.3)	0.399
Body mass index, kg/m^2^	26.6 (5.3)	27.4 (5.7)	26.1 (5.0)	23.6 (3.3)	0.192
Appendicular muscle mass, kg	19.5 (4.4)	19.8 (4.4)	19.4 (3.8)	18.5 (7.4)	0.776
Skeletal muscle mass index, kg/m^2^	7.3 (1.0)	7.4 (1.0)	7.3 (0.9)	6.8 (1.3)	0.375
Insulin, *n* (%)	18 (19.8)	8 (17.4)	8 (20.5)	2 (33.3)	0.646
SGLT2 inhibitor, *n* (%)	26 (28.6)	14 (30.4)	11 (28.2)	1 (16.7)	0.780
Hypertension, *n* (%)	57 (62.6)	31 (67.4)	23 (59.0)	3 (50)	0.584
Dyslipidemia*, n* (%)	63 (69.2)	36 (78.3)	23 (59.0)	4 (66.7)	0.157
Smoker*, n* (%)	17 (18.7)	7 (15.2)	9 (23.1)	1 (16.7)	0.646
Exerciser*, n* (%)	35 (38.5)	16 (34.8)	17 (43.6)	2 (33.3)	0.683
Alcohol intake*, n* (%)	12 (13.2)	6 (13.0)	5 (12.8)	1 (16.7)	0.966
HbA1c, mmol/mol	61.4 (19.1)	60.9 (14.8)	63.0 (23.9)	54.6 (12.9)	0.600
HbA1c, %	7.8 (1.7)	7.7 (1.4)	7.9 (2.2)	7.2 (1.2)	0.600
Plasma glucose, mmol/L	8.6 (2.9)	8.4 (2.4)	9.1 (3.5)	7.5 (2.4)	0.302
Total energy intake, kcal/day	1761 (571)	1810 (6584)	1726 (576)	1608 (450)	0.637
Total energy intake, kcal/kg IBW/day	30.4 (9.5)	31.2 (9.8)	29.8 (9.4)	28.2 (10.0)	0.691
Protein intake, g/ day	67.3 (20.8)	69.1 (22.3)	66.2 (19.6)	61.4 (17.6)	0.632
Protein intake, % Energy	15.6 (2.9)	15.5 (2.9)	15.7 (3.0)	15.4 (1.4)	0.935
Fat intake, g/ day	54.7 (18.8)	57.1 (20.0)	53.3 (17.9)	45.1 (12.3)	0.290
Fat intake, % Energy	28.2 (5.9)	28.5 (5.8)	28.3 (6.2)	25.7 (4.2)	0.554
Carbohydrate intake, g/ day	226.6 (83.1)	235.8 (95.2)	217.3 (68.2)	215.6 (75.1)	0.565
Carbohydrate intake, % Energy	51.6 (8.2)	51.9 (8.2)	50.9 (8.1)	53.4 (8.6)	0.733
SMI decrease, kg/m^2^/year	0.004 (0.023)	0.005 (0.022)	0.005 (0.023)	−0.008 (0.024)	0.385
Rate of SMI decrease, %	0.60 (3.72)	0.81 (3.38)	0.69 (3.96)	−1.66 (4.55)	0.306
Follow up duration, years	1.6 (0.6)	1.5 (0.5)	1.6 (0.5)	2.3 (0.9)^†^^‡^	0.008
**Age ≥65 years old**	**All** ***n*** **= 193**	**Fast**, ***n*** **= 78**	**Normal**, ***n*** **= 74**	**Slow**, ***n*** **= 41**	***p***
Age, years	72.2 (5.2)	72.1 (5.2)	71.4 (5.0)	74.0 (5.2)	0.037
Men, *n* (%)	109 (56.5)	45 (57.7)	36 (48.7)	28 (68.3)	0.121
Duration of diabetes, years	15.9 (10.0)	16.5 (10.3)	15.0 (10.4)	16.4 (8.8)	0.623
Family history of diabetes, *n* (%)	81 (42.0)	37 (47.4)	24 (32.4)	20 (48.8)	0.105
Body mass index, kg/m^2^	23.8 (3.9)	24.1 (3.4)	24.2 (4.4)	22.5 (3.6)	0.046
Appendicular muscle mass, kg	17.7 (3.9)	18.1 (3.9)	17.3 (3.9)	17.8 (3.6)	0.375
Skeletal muscle mass index, kg/m^2^	6.9 (1.0)	6.9 (1.0)	6.8 (1.0)	6.8 (0.9)	0.679
Insulin, *n* (%)	51 (26.4)	23 (29.5)	17 (23.0)	11 (26.8)	0.659
SGLT2 inhibitor, *n* (%)	25 (13.0)	13 (16.7)	7 (9.5)	5 (12.2)	0.412
Hypertension, *n* (%)	137 (71.0)	55 (70.5)	53 (71.6)	29 (70.7)	0.988
Dyslipidemia, *n* (%)	134 (69.4)	51 (65.4)	53 (71.6)	30 (73.2)	0.595
Smoker, *n* (%)	27 (14.0)	14 (18.0)	7 (9.5)	6 (14.6)	0.318
Exerciser, *n* (%)	109 (56.5)	48 (61.5)	38 (51.4)	23 (56.1)	0.448
Alcohol intake, *n* (%)	19 (9.8)	4 (5.1)	6 (8.1)	9 (22.0)	0.011
HbA1c, mmol/mol	55.3 (10.8)	55.5 (11.3)	55.0 (11.2)	55.2 (9.0)	0.953
HbA1c, %	7.2 (1.0)	7.2 (1.0)	7.2 (1.0)	7.2 (0.8)	0.953
Plasma glucose, mmol/L	8.2 (2.9)	8.3 (3.5)	8.0 (2.1)	8.6 (2.8)	0.585
Total energy intake, kcal/day	1748 (602)	1799 (672)	1719 (602)	1702 (447)	0.619
Total energy intake, kcal/kg IBW/day	30.9 (10.1)	31.3 (10.9)	31.1 (10.4)	29.8 (8.0)	0.699
Protein intake, g/ day	75.6 (30.5)	75.2 (29.8)	78.2 (34.0)	71.8 (25.0)	0.549
Protein intake, % Energy	17.4 (3.6)	17.0 (3.4)	18.1 (3.9)	16.7 (3.3)	0.076
Fat intake, g/ day	55.9 (22.2)	55.5 (19.5)	57.5 (26.3)	53.6 (19.1)	0.664
Fat intake, % Energy	29.0 (6.5)	28.6 (6.5)	29.8 (6.5)	28.1 (6.4)	0.359
Carbohydrate intake, g/ day	217.7 (82.9)	234.2 (103.5)	206.4 (65.6)	206.6 (60.4)	0.075
Carbohydrate intake, % Energy	50.1 (9.1)	51.4 (8.7)	49.0 (9.2)	49.5 (9.6)	0.246
SMI decrease, kg/m^2^/year	0.005 (0.021)	−0.001 (0.020)	0.010 (0.023)^†^	0.009 (0.017)^†^	0.003
Rate of SMI decrease, %	0.88 (3.75)	−0.24 (3.79)	1.69 (3.94)^†^	1.55 (2.73)^†^	0.003
Follow up duration, years	1.7 (0.7)	1.8 (0.7)	1.7 (0.7)	1.8 (0.6)	0.410

*Data was expressed as mean (standard deviation) or number (%). The difference between group was evaluated by one–way analysis of variance and Tukey–Kramer test, or chi–squared test. SMI, skeletal muscle mass; SGLT, sodium–glucose cotransporter; IBW, ideal body weight*.

† *p < 0.05 vs. fast speed eating and*

‡ *p < 0.05 vs. normal speed eating by Tukey–Kramer test*.

In patients aged <65 years, the follow–up duration of the slow–speed eating group was significantly longer than that of the fast– and normal–speed eating groups (*p* < 0.05), and there were no significant differences in total energy intake and protein intake among the groups. In patients aged ≥65 years, the SMI decrease and the rate of SMI decrease in the slow– and normal–speed eating groups were lower than those in the fast–speed eating group, with significant differences (*p* < 0.05) (Table 1).

The relationship between eating speed and SMI decrease (kg/m^2^/year) or rate of SMI decrease (%) adjusted for age, sex, smoking, exercise, alcohol, use of insulin and use of SGLT2 inhibitors is shown in Table 2. In patients aged <65 years, there were no statistically significant differences in SMI decrease or rate of SMI decrease among the groups. On the other hand, in patients aged ≥65 years, SMI decrease of fast eating groups (−0.006 [95% CI −0,014 to 0.003] and rate of SMI decrease of fast eating groups (−1.08 [95% CI −2.52 to 0.36]) were lower than those of slow speed eating after adjusting for covariates, and there were statistically significant differences.

**Table 2:** Relationship between eating speed and SMI decrease (kg/m^2^/year) or rate of SMI decrease (%).

**Age <65 years old**	**SMI decrease (kg/m** ^**2**^ **/year)**	**Rate of SMI decrease (%)**
Fast	0.005 (−0.005–0.015)	0.67 (−0.96–2.31)
Normal	0.002 (−0.007–0.012)	0.58 (−1.34–1.87)
Slow	−0.009 (−0.027–0.009)	−1.84 (−4.85–1.17)
**Age ≥65 years old**	**SMI decrease (kg/m** ^**2**^ **/year)**	**Rate of SMI decrease (%)**
Fast	−0.006 (−0.014–0.003)	−1.08 (−2.52–0.36)
Normal	0.005 (−0.003–0.014)^†^	0.85 (−0.66–2.35)^†^
Slow	0.006 (−0.003–0.014)^†^	0.93 (−0.61–2.46)^†^

*Values for outcome variables are geometric means and 95% CI. SMI, Skeletal muscle mass index. Adjusted for age, sex, smoking, exercise, alcohol, insulin, sodium–glucose cotransporter inhibitor, total energy intake (kcal/kg ideal body weight/day), and protein intake (% Energy)*.

† *p < 0.05 vs. fast speed eating by Tukey–Kramer test*.

**Figure 2:**
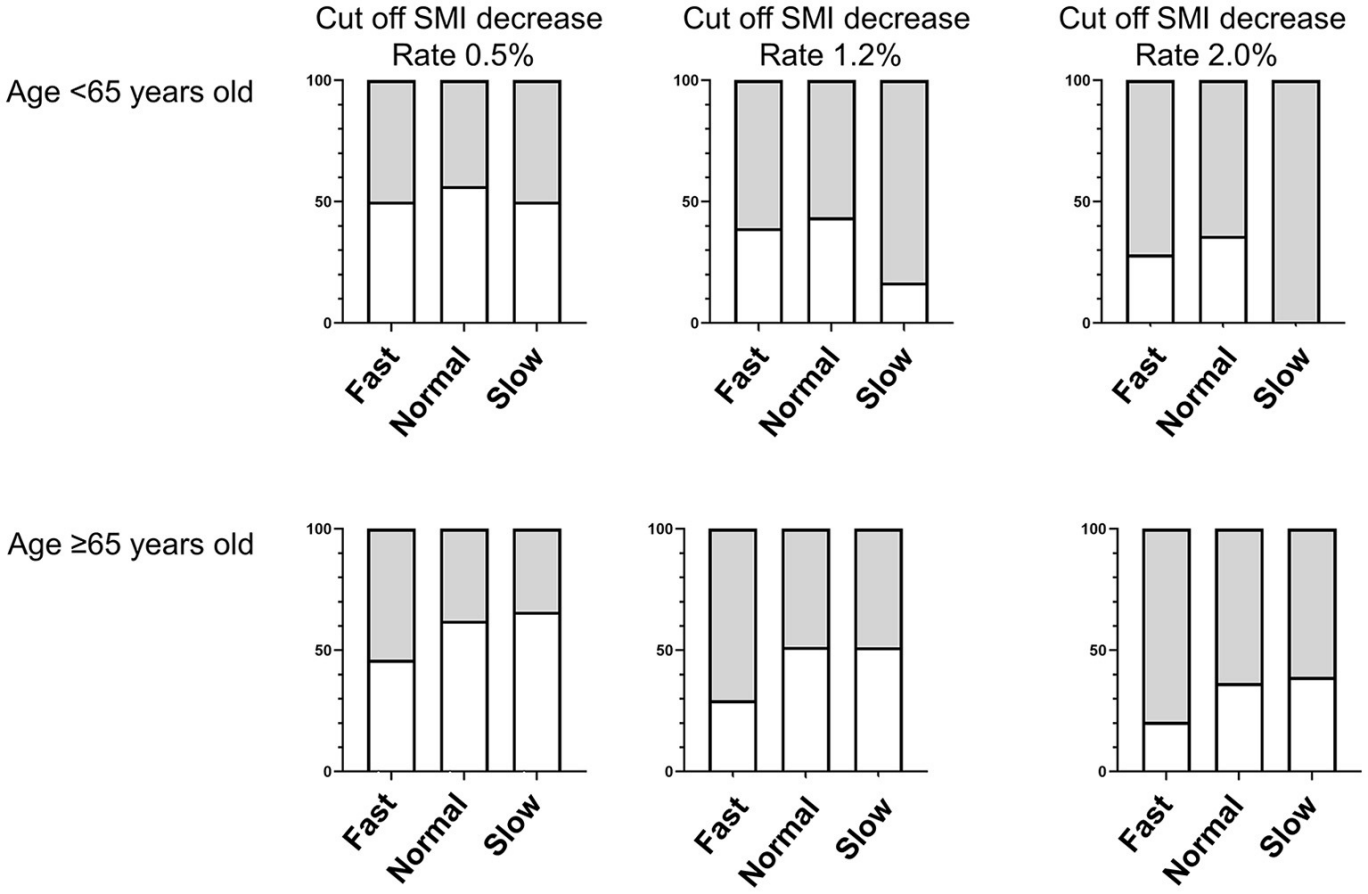
Proportions of muscle mass loss among fast, normal, and slow speed eating by age (Cut–off levels of the rate of SMI decrease Rate 0.5%, 1.2% and 2.0%). Gray square represents the proportion of non–sarcopenia and white square represents the proportion of sarcopenia.

The relationship between eating speed and incident muscle mass loss is presented in Table 3 and Figure 2. In patients aged <65 years, alcohol intake (odds ratio 0.11, [95% CI 0.02 to 0.67], *p* = 0.016), total energy intake (odds ratio 0.90, [95% CI 0.85 to 0.96], *p* = 0.002), and protein intake (odds ratio 0.77, [95% CI 0.62 to 0.96], *p* = 0.019) were considered to have a lower risk of incident muscle mass loss but not in patients aged ≥65 years. Furthermore, the adjusted odds ratios of the incident muscle loss of fast speed eating were 0.42 (95% CI 0.18 to 0.98, *p* = 0.044) compared with slow speed eating, in patients aged ≥65 years.

**Table 3:** Relationship between eating speed and the incident muscle mass loss.

**Age <65 years old**	**Model 1**		**Model 2**		**Model 3**	
	**Odds ratio (95% CI)**	***p*** **–value**	**Odds ratio (95% CI)**	***p*** **–value**	**Odds ratio (95% CI)**	***p*** **–value**
Age (year)	—	—	0.96 (0.90–1.02)	0.204	0.97 (0.90–1.03)	0.310
Men	—	—	0.69 (0.23–2.06)	0.502	0.78 (0.25–2.42)	0.671
HbA1c (%)	—	—	0.70 (0.50–0.98)	0.038	0.68 (0.47–0.97)	0.032
Insulin usage (yes)	—	—	1.14 (0.31–4.20)	0.846	1.26 (0.33–4.79)	0.737
SGLT2 inhibitor (yes)	—	—	1.69 (0.54–5.31)	0.368	1.62 (0.51–5.15)	0.415
Smoking (yes)	—	—	1.54 (0.41–5.87)	0.523	1.47 (0.39–5.61)	0.570
Exercise (yes)	—	—	1.03 (0.35–3.02)	0.960	0.97 (0.33–2.87)	0.950
Alcohol (yes)	—	—	0.13 (0.02–0.75)	0.022	0.11 (0.02–0.67)	0.016
Total energy intake (kcal/kg IBW/day)	—	—	0.91 (0.86–0.97)	<0.001	0.90 (0.85–0.96)	0.002
Protein intake (% Energy)	—	—	0.78 (0.63–0.96)	0.015	0.77 (0.62–0.96)	0.019
Body mass index (kg/m^2^)	—	—	—	—	1.06 (0.95–1.19)	0.265
Eating speed						
Fast	1.00 (0.18–5.48)	1.000	1.53 (0.20–11.6)	0.680	1.31 (0.17–10.0)	0.796
Normal	1.29 (0.23–7.23)	0.769	2.17 (0.27–17.5)	0.468	1.95 (0.24–15.6)	0.529
Slow	Reference	—	Reference	—	Reference	—
**Age ≥65 years old**	**Model 1**		**Model 2**		**Model 3**	
	**Odds ratio (95% CI)**	***p*** **–value**	**Odds ratio (95% CI)**	***p*** **–value**	**Odds ratio (95% CI)**	***p*** **–value**
Age (year)	—	—	0.95 (0.89–1.01)	0.122	0.95 (0.89–1.01)	0.098
Men	—	—	1.39 (0.70–2.75)	0.337	1.40 (0.71–2.77)	0.328
HbA1c (%)	—	—	1.30 (0.93–1.82)	0.122	1.29 (0.92–1.80)	0.140
Insulin usage (yes)	—	—	1.26 (0.60–2.64)	0.535	1.26 (0.60–2.63)	0.539
SGLT2 inhibitor (yes)	—	—	1.55 (0.60–4.01)	0.369	1.64 (0.62–4.35)	0.318
Smoking (yes)	—	—	0.52 (0.20–1.33)	0.174	0.51 (0.20–1.32)	0.166
Exercise (yes)	—	—	0.96 (0.52–1.77)	0.887	0.95 (0.51–1.76)	0.873
Alcohol (yes)	—	—	0.87 (0.28–2.66)	0.806	0.85 (0.28–2.63)	0.784
Total energy intake (kcal/kg IBW/day)	—	—	0.98 (0.95–1.01)	0.289	0.98 (0.95–1.01)	0.270
Protein intake (% Energy)	—	—	0.97 (0.88–1.06)	0.447	0.97 (0.88–1.06)	0.439
Body mass index (kg/m^2^)	—	—	—	—	0.97 (0.89–1.06)	0.555
Eating speed						
Fast	0.44 (0.20–0.97)	0.043	0.40 (0.17–0.93)	0.034	0.42 (0.18–0.98)	0.044
Normal	0.85 (0.38–1.89)	0.694	0.82 (0.35–1.95)	0.656	0.82 (0.36–2.03)	0.715
Slow	Reference	—	Reference	—	Reference	—

*Incident muscle mass loss was defined as the rate of skeletal muscle mass index decrease (%) ≥0.5%. SGLT, Sodium–glucose cotransporter, IBW; IBW, ideal body weight*.

In addition, the analysis results using different cutoff levels for muscle mass loss for the rates of SMI decrease of ≥1.2% and ≥2.0% in patients aged ≥65 years are shown in Supplementary Table 1 and Figure 2. The risk of the incident muscle mass loss due to fast speed eating (cutoff point: rate of SMI decrease of ≥1.2%, odds ratio 0.38 [95% CI 0.16 to 0.88], *p* = 0.024, and cutoff point: rate of SMI decrease of ≥2.0%, odds ratio 0.32 [95% CI 0.13 to 0.81], *p* = 0.015) was lower than that due to slow speed eating.

## Discussion

This prospective study showed that slow–speed eating had the potential to promote SMI decrease and incident muscle mass loss in patients aged ≥65 years with T2DM. Furthermore, alcohol consumption, protein intake and total energy intake were considered low risk factors for muscle mass loss in patients aged <65 years with T2DM.

In general, muscle mass decreases with age (27), and adequate energy and protein intake are important for maintaining muscle mass (12, 13), which aids in sarcopenia prevention. Furthermore, lack of energy leads to a decrease in muscle mass, especially in elderly patients with T2DM (12). In this study, there was a significant difference in muscle mass loss depending on eating speed in patients aged ≥65 years with T2DM, even after adjusting for total energy intake and total protein intake.

Insulin stimulates protein synthesis, and defects in insulin signaling lead to decreased muscle mass (27). By contrast, fast–speed eating can cause elevation in the levels of postprandial blood glucose and FPG (18), which leads to increased insulin secretion and stimulation of protein synthesis. Additionally, slower eating speed and longer chewing time increase diet–induced thermogenesis (20, 21). This can lead to loss of muscle mass while preventing obesity. Furthermore, eating slowly increases anorexigenic gut hormones such as peptide YY and GLP−1 (18, 19). GLP−1 is a hormone that not only decreases appetite and body weight (19) but also maintains muscle mass (36). The reasons for this are thought to be GLP−1's various effects: increasing glucose sensitivity, neogenesis, proliferation, hypertrophy, and transcription of pro–insulin, while reducing apoptosis of beta cells (37–40). Therefore, slow eating speed might have a greater impact on decreasing appetite than on increasing muscle mass. For these reasons, although there were no differences in total energy and protein intakes, muscle mass of patients aged ≥65 years with T2DM decreased in the slow–speed eating group.

In addition, there is a relationship between sarcopenia and swallowing function in elderly people (22), and decreased swallowing function affects the eating speed. Therefore, it is possible that eating speed was slower in patients with reduced muscle mass.

However, this study showed that eating speed did not affect muscle mass loss in patients aged <65 years old. Younger people maintain insulin secretion (41) and anabolic effects (42); therefore, it is possible that muscle mass did not decrease.

The present study also reported that slow–speed eating was related to poor nutrition (17). By contrast, fast eating speed is related to obesity, T2DM, and NAFLD (14–16). The reasons for this may be as follows: slow eating speed leads to lower energy intake (43), but fast eating speed is related to increased energy intake (14, 43, 44). Furthermore, fast speed eating is associated with obesity, reducing energy consumption after meals, phosphorylation of Akt because of postprandial hyperglycemia and hyperinsulinemia (16). However, in this study, there was no significant difference between eating speed, total energy intake, and protein intake, regardless of age. Previous reports have shown that obese patients with T2DM under–report their food intake (45, 46), and this might have had an effect on total energy and protein intakes.

Alcohol consumption causes an increase in cortisol (47) levels and a decrease in mTOR signaling (48, 49), which leads to muscle mass loss (50). By contrast, alcohol intake may be associated with a lower incidence of frailty (51). Modest alcohol consumption leads to higher serum concentrations of dehydroepiandrosterone (DHEA) and testosterone (52, 53). These hormones increase loss of muscle mass (54, 55). In patients aged <65 years, alcohol intake may increase DHEA and testosterone levels and prevent muscle mass loss.

As the number of elderly patients with T2DM increases, the prevalence of sarcopenia also increases. This leads to increased mortality, which is a serious health problem. This study showed that slow–speed eating was associated with muscle mass loss. Therefore, it is important to consider eating speed in elderly patients with T2DM.

This study had some limitations. First, a self–reported questionnaire was used for assessment of eating speed and it was not measured directly. In addition, no clear definition of slow speed eating was showed by using seconds or minutes per specific volume/food type. Thus, there is a possibility that eating speed might have differed from the actual speed of eating. Second, body composition analysis was performed by a BIA for evaluating skeletal muscle mass, although the gold standard method was DEXA. A previous study has reported that BIA underestimated lean mass and overestimates fat mass, compared to DEXA (22). Third, we defined exercises from self–reported questionaries and have not evaluated the actual physical activity by objective criteria such as metabolic equivalents (METs). Fourth, we collected questionnaires only one time at the first time and did not re–evaluate. Lastly, this study included only Japanese patients with T2D; hence, generalization of the results to other groups is still unknown.

## Conclusion

Slow–speed eating was associated with muscle mass loss in older patients with T2DM; therefore, close attention needed to the eating speed when treating older patients with T2DM.

## Data Availability Statement

The raw data supporting the conclusions of this article will be made available by the authors, without undue reservation.

## Ethics Statement

The studies involving human participants were reviewed and approved by the Ethics Committee of KPUM (No. RBMR–E−466–5). The patients/participants provided their written informed consent to participate in this study.

## Author Contributions

GK interpreted the data and wrote the manuscript. YH conceived and designed the study, acquired, analyzes, interpreted the data, and contributed discussion. FT acquired and interpreted the data and contributed discussion. AK, MH, and RS conceived and designed the work, acquired the data, and contributed discussion. TO, NK, HO, NN, SM, TS, EU, MY, and MA acquired the data and contributed discussion. MF conceived and designed the work, acquired and interpreted the data, and revised the manuscript. All authors have read and agreed to the published version of the manuscript.

## Conflict of Interest

YH has received honoraria from Kowa Co., Ltd., Sanofi K.K., Takeda Pharmaceutical Co., Ltd., Terumo Corporation, and Chugai Pharmaceutical Co., Ltd., outside the submitted work. HO received grant support from the Japan Society for the Promotion of Science and received personal fees from MSD K.K., Mitsubishi Tanabe Pharma Corporation, Sumitomo Dainippon Pharma Co., Ltd., Novo Nordisk Pharma Ltd., Daiichi Sankyo Co., Ltd., Eli Lilly Japan K.K., Kyowa Hakko Kirin Company Ltd., Kissei Pharmaceutical Co., Ltd., Takeda Pharmaceutical Co., Ltd., Kowa Pharmaceutical Co., Ltd., Ono Pharmaceutical Co., Ltd., and Sanofi K.K., outside the submitted work. NN received grant support from Japan Society for the Promotion of Science, the Japan Food Chemical Research Foundation, and received personal fees from Kowa Pharmaceutical Co., Ltd., and Novo Nordisk Pharma Ltd., outside the submitted work. TO has received honoraria from MSD K.K., Sumitomo Dainippon Pharma Co., Ltd., Novo Nordisk Pharma Ltd., DAIICHI SANKYO COMPANY, LIMITED, Eli Lilly Japan K.K., T Takeda Pharmaceutical Co., Ltd., Nippon Boehringer Ingelheim Co., Ltd., Mitsubishi Tanabe Pharma Corporation, Kyowa Kirin Co., Ltd., Kowa Co., Ltd., ONO PHARMACEUTICAL Co., Ltd., TOA EIYO Corporation, and AstraZeneca K.K., grant from Combi Corporation, outside the submitted work. TS has received personal fees from Eli Lilly Japan K.K., Mitsubishi Tanabe Pharma Co., Kowa Pharma Co., Ltd., Astellas Pharma Inc., Takeda Pharma Co., Ltd., Sanofi K.K., Taisho Toyama Pharma Co., Ltd., Kyowa Hakko Kirin Co., Ltd., Kissei Pharma Co., Ltd., MSD K.K., Novo Nordisk Pharma Ltd., Ono Pharma Co., Ltd., outside the submitted work. EU received grant support from the Japanese Study Group for Physiology and Management of Blood Pressure, the Astellas Foundation for Research on Metabolic Disorders, the Japan Society for the Promotion of Science, Mishima Kaiun Memorial Foundation, and received personal fees from Nippon Boehringer Ingelheim Co., Ltd., Mitsubishi Tanabe Pharma Corporation, Daiichi Sankyo Co., Ltd., Takeda Pharmaceutical Co., Ltd., MSD K.K., Kyowa Hakko Kirin Co., Ltd., Sumitomo Dainippon Pharma Co., Ltd., Kowa Pharmaceutical Co., Ltd., Novo Nordisk Pharma Ltd., Ono Pharmaceutical Co., Ltd., Taisho Pharmaceutical Co., Ltd., AstraZeneca K.K., and Sanofi K.K., outside the submitted work. Donated Fund Laboratory of Diabetes therapeutics is an endowment department, supported with an unrestricted grant from Ono Pharmaceutical Co., Ltd., Taiyo Kagaku Co., Ltd., and Taisho Pharmaceutical Co., Ltd., outside the submitted work. MA received personal fees from Takeda Pharmaceutical Co., Ltd., Abbott Japan Co., Ltd., Sumitomo Dainippon Pharma Co., Ltd., Kowa Pharmaceutical Co., Ltd., Novo Nordisk Pharma Ltd., Ono Pharmaceutical Co., Ltd., AstraZeneca K.K., and Chugai Pharmaceutical Co., Ltd., outside the submitted work. MH received grants from AstraZeneca K.K., Ono Pharma Co., Ltd., Oishi Kenko inc., Yamada Bee Farm, Nippon Boehringer Ingelheim Co., Ltd., and received personal fees from AstraZeneca K.K., Ono Pharma Co., Ltd., Eli Lilly, Japan, Sumitomo Dainippon Pharma Co., Ltd., Daiichi Sankyo Co., Ltd., Mitsubishi Tanabe Pharma Corp., Sanofi K.K., and Kowa Pharma Co., Ltd., outside the submitted work. MY received personal fees from MSD K.K., Sumitomo Dainippon Pharma Co., Ltd., Kowa Co., Ltd., AstraZeneca PLC, Takeda Pharmaceutical Co., Ltd., Kyowa Hakko Kirin Co., Ltd., Daiichi Sankyo Co., Ltd., Kowa Pharmaceutical Co., Ltd., and Ono Pharmaceutical Co., Ltd., outside the submitted work. MF received grants from Ono Pharma Co., Ltd., Oishi Kenko inc., Yamada Bee Farm, Nippon Boehringer Ingelheim Co., Ltd., Kissei Pharma Co., Ltd., Mitsubishi Tanabe Pharma Corp., Daiichi Sankyo Co., Ltd., Sanofi K.K., Takeda Pharma Co., Ltd., Astellas Pharma Inc., MSD K.K., Kyowa Kirin Co., Ltd., Sumitomo Dainippon Pharma Co., Ltd., Kowa Pharma Co., Ltd., Novo Nordisk Pharma Ltd., Sanwa Kagagu Kenkyusho Co., Ltd., Eli Lilly, Japan, K.K., Taisho Pharma Co., Ltd., Terumo Corp., Tejin Pharma Ltd., Nippon Chemiphar Co., Ltd., Abbott Japan Co., Ltd., and Johnson and Johnson K.K. Medical Co., TERUMO CORPORATION, and received personal fees from Nippon Boehringer Ingelheim Co., Ltd., Kissei Pharma Co., Ltd., Mitsubishi Tanabe Pharma Corp., Daiichi Sankyo Co., Ltd., Sanofi K.K., Takeda Pharma Co., Ltd., Astellas Pharma Inc., MSD K.K., Kyowa Kirin Co., Ltd., Sumitomo Dainippon Pharma Co., Ltd., Kowa Pharma Co., Ltd., Novo Nordisk Pharma Ltd., Ono Pharma Co., Ltd., Sanwa Kagaku Kenkyusho Co., Ltd., Eli Lilly Japan K.K., Taisho Pharma Co., Ltd., Bayer Yakuhin, Ltd., AstraZeneca K.K., Mochida Pharma Co., Ltd., Abbott Japan Co., Ltd., Teijin Pharma Ltd., Arkray Inc., Medtronic Japan Co., Ltd., and Nipro Corp., TERUMO CORPORATION, outside the submitted work. These sponsors were not involved in the study design, collection, analysis, or interpretation of data, in the writing of this manuscript, or in the decision to submit the article for publication. The remaining authors declare that the research was conducted in the absence of any commercial or financial relationships that could be construed as a potential conflict of interest.

## Publisher's Note

All claims expressed in this article are solely those of the authors and do not necessarily represent those of their affiliated organizations, or those of the publisher, the editors and the reviewers. Any product that may be evaluated in this article, or claim that may be made by its manufacturer, is not guaranteed or endorsed by the publisher.
