# Supplementary material for: Impact of Eating Speed on Muscle Mass in Older Patients With Type 2 Diabetes: A Prospective Study of KAMOGAWA–DM Cohort
Source: Front Nutr. 2022 Jun 23;9:919124. doi: 10.3389/fnut.2022.919124 (PMC9260176; doi:10.3389/fnut.2022.919124)
Supplement: Supplementary file 1 [file Table_1.docx]

**Supplemental Table. Relationship between eating speed and the incident muscle mass loss**

| Age ≥65 years old | Rate 1.2% | | Rate 2.0% | |
| --- | --- | --- | --- | --- |
|  | Odds ratio (95% CI) | *p* value | Odds ratio (95% CI) | *p* value |
| Age (year) | 0.98 (0.92 – 1.04) | 0.420 | 0.96 (0.90 – 1.03) | 0.256 |
| Men | 1.31 (0.66 – 2.59) | 0.436 | 0.98 (0.48 – 2.01) | 0.954 |
| HbA1c (%) | 1.35 (0.97 – 1.89) | 0.078 | 1.33 (0.94 – 1.87) | 0.106 |
| Insulin usage (yes) | 0.77 (0.37 – 1.62) | 0.495 | 0.87 (0.40 – 1.91) | 0.732 |
| SGLT2 inhibitor (yes) | 0.98 (0.37 – 2.58) | 0.968 | 1.66 (0.63 – 4.42) | 0.309 |
| Smoking (yes) | 1.11 (0.43 – 2.89) | 0.830 | 1.06 (0.38 – 2.98) | 0.906 |
| Exercise (yes) | 0.76 (0.41 – 1.41) | 0.378 | 0.77 (0.40 – 1.49) | 0.437 |
| Alcohol (yes) | 0.62 (0.20 – 1.94) | 0.413 | 0.41 (0.11 – 1.58) | 0.198 |
| Total energy intake (kcal/kg IBW/day) | 0.98 (0.95 – 1.01) | 0.184 | 0.99 (0.96 – 1.02) | 0.528 |
| Protein intake (% Energy) | 0.95 (0.87 – 1.05) | 0.321 | 1.00 (0.91 – 1.11) | 0.960 |
| Body mass index (kg/m^2^) | 0.98 (0.90 – 1.07) | 0.655 | 0.97 (0.88 – 1.07) | 0.581 |
| Eating speed |  |  |  |  |
| Fast | 0.38 (0.16 – 0.88) | 0.024 | 0.32 (0.13 – 0.81) | 0.015 |
| Normal | 1.04 (0.45 – 2.40) | 0.930 | 0.75 (0.31 – 1.80) | 0.522 |
| Slow | Reference | ― | Reference | ― |

The analyses were conducted when the incident muscle mass loss was defined as the rate of skeletal muscle mass index decrease (%) ≥1.2% and ≥ 2.0%. SGLT, Sodium-glucose cotransporter, IBW; IBW, ideal body weight.
